# Parent psychological distress and parent-child relationships two years into the COVID-19 pandemic: Results from a Canadian cross-sectional study

**DOI:** 10.1371/journal.pone.0292670

**Published:** 2023-10-17

**Authors:** Kimberly C. Thomson, Emily Jenkins, Randip Gill, Katherine G. Hastings, Chris G. Richardson, Monique Gagné Petteni, Corey McAuliffe, Anne M. Gadermann

**Affiliations:** 1 Children’s Health Policy Centre, Faculty of Health Sciences, Simon Fraser University, Burnaby, Canada; 2 Human Early Learning Partnership, School of Population and Public Health, University of British Columbia, Vancouver, Canada; 3 Centre for Advancing Health Outcomes, Providence Health Care Research Institute, Vancouver, British Columbia, Canada; 4 School of Nursing, University of British Columbia, Vancouver, Canada; 5 School of Population and Public Health, University of British Columbia, Vancouver, Canada; University of Central Florida, UNITED STATES

## Abstract

**Background:**

Mental health impacts of the COVID-19 pandemic have not been felt equally within populations. Parents with children living at home were early on identified as a population at heightened mental health risk, with concerns about the potential long-term impacts of the pandemic on parents’ mental health, family functioning, and children’s well-being. This study investigates impacts of the pandemic on parents’ psychological distress, contextual sources of distress, and associations with family functioning nearly two years into the pandemic.

**Methods and findings:**

Data were drawn from a national cross-sectional survey of adults living in Canada in November and December 2021 that was representative by age, gender, household income, and region. Parents with children < 18 years old living at home (N = 553) reported their experiences of psychological distress, pandemic-related stressors, coping mechanisms, and family functioning (changes in parent-child interactions, children’s anxiety). Chi-square tests, logistic regression, and linear regression analyses examined sociodemographic inequities in parents’ levels of psychological distress, sources and mitigating mechanisms of distress, and associations between psychological distress and family functioning. Nearly two years into the pandemic, parents with children at home reported nearly double pre-pandemic population estimates of moderate to severe psychological distress. Psychological distress was more frequently reported among parents with pre-existing mental health conditions, disabilities, and financial stressors. Parents with greater psychological distress reported increases in negative parent–child interactions due to the pandemic and higher anxiety among their children.

**Conclusions:**

This study identifies sustained negative impacts of the pandemic on parents’ mental health and family functioning in Canada nearly two years into the pandemic, despite high vaccine uptake and declining infection rates. Disparities in financial stress, social support structures, and pre-existing mental health were identified as underlying sources of psychological distress. These results highlight that meaningful responses to promote mental health among parents and families must address social and structural inequities.

## Introduction

Parents with children living at home have been identified as a population at heightened risk of worsened mental health and psychological distress due to the COVID-19 pandemic [1–4]. In addition to the stress and uncertainty experienced in the general population, parents have encountered unique stressors including school and child care closures, financial stress of supporting the household, worrying about their children’s well-being and education, and strains on their relationships with their children [1–4]. Early into the pandemic, mental health experts voiced concerns regarding the long-term population mental health impacts of social disruptions, economic uncertainty, and extended exposure to stress [5, 6]. Research identified that parent stress increased substantially during the initial wave of the COVID-19 pandemic in May 2020 and had not returned to pre-pandemic levels by 2022 [7–9]. This research also consistently identified social gradients in the severity of mental health impacts among population groups, with women, parents with lower incomes, single parents, and parents with younger children reporting higher levels of psychological distress than other groups [5, 10].

While there is substantial research documenting the ways that the pandemic has impacted parent mental health, there is also emerging evidence that pandemic-related stress has negatively impacted parent-child interactions, with reports of pandemic-related increases in family chaos and conflict [11–14]. The family stress model proposed by Conger et al. [15] articulates a pathway between family stressors and conflict on adolescent distress, including internalizing (i.e., emotional or psychological challenges that manifest as inward-facing distress, such as depression and anxiety) and externalizing behaviours (i.e., emotional or psychological challenges that manifest as outward-facing distress, such as physical aggression) [15]. For example, stress arising from parental financial challenges can affect adolescent development indirectly through parental hostility, which in turn, increases the risk of adolescent internalizing and externalizing behaviours. Adverse economic conditions brought on by the pandemic have increased financial conflict in many families, with additional stressors such as prolonged social isolation, disruptions to daily routine, and fears about the virus affecting health potentially compounding strains on parent-child relationships [1]. Some research suggests that children have experienced elevated levels of depression and anxiety during the pandemic [16, 17], and the relationship between these mental health effects and parental distress warrants further investigation. To date, most research literature has described the initial mental health impacts of the pandemic on family functioning, leaving substantial gaps in knowledge about the sustained impacts on parent-child relationships beyond the early months of the pandemic [18, 19]. If left unaddressed, some experts have warned of the potential for ‘family scarring’, or prolonged, intertwined individual mental health and family relationship issues over time [13].

In prior research that informed the current study, we identified that within the first year of the pandemic, parents in Canada who reported pandemic-related stressors including financial concerns and relationship challenges also reported increases in both negative and positive interactions with their children [2, 8]. The present study extends these findings, examining the impacts of the pandemic on parents’ psychological distress using data from nearly two years into the pandemic. The study objectives were (1) to investigate the prevalence of psychological distress among parents, as well as the sources of parents’ psychological distress and mitigating factors; and (2) to examine the associations between parents’ psychological distress, changes in positive and negative parent-child interactions, and perceptions of their children’s anxiety.

## Methods

### Procedure

Data were collected as part of a multi-round cross-sectional survey of adults living in Canada entitled, “Assessing the Impacts of COVID-19 on Mental Health,” conducted as a partnership between the University of British Columbia and the Canadian Mental Health Association. The current investigation draws on the fourth round of data, which were collected in November and December of 2021. This time corresponded with a period of relative safety and stability immediately prior to the rapid proliferation of the Omicron variant across the country. During this time, employment and labour output in Canada had recovered to pre-pandemic levels and 80% of eligible Canadians were fully vaccinated [20].

Participants were recruited from a national Canadian survey panel of approximately 125,000 adults maintained by polling vendor Maru/Matchbox. Panel members were randomly invited based on national census informed stratifications by age, gender, household income and region, to generate a nationally representative sample according to these sociodemographic characteristics. Maru/Matchbox recruited panel participants through direct email and engaged affiliate community partners to increase inclusion of populations that may be difficult to reach via the Internet, including older adults and racialized populations [21]. This sampling method resulted in a response-to-invitation ratio of 30%, yielding a total sample of 3030 adult participants. For our present analyses, the sample was restricted to participants identifying as parents/guardians with children <18 years old currently living at home (N = 553). All participants completed an online consent process prior to beginning the survey and were provided with a small honorarium through Maru/Matchbox to compensate for their time. Ethics approval was provided by the Behavioural Research Ethics Board at the University of British Columbia (H20-01273).

### Measures

Survey items were informed by a monitoring survey first commissioned by the Mental Health Foundation in March 2020 (London, UK) and informed through consultation with people with lived experience of mental health conditions [22]. Our research team, in consultation with collaborators from the Canadian Mental Health Association, subsequently modified the survey to reflect the Canadian context. In this fourth round of data collection, we added two previously-validated scales to assess adults’ psychological distress and parents’ perceptions of their children’s anxiety. Survey items used in this analysis are provided in the S1 Table.

***Parent background characteristics*** included parent age, gender, pre-existing mental health condition(s) (i.e., onset prior to pandemic), and disability.

***Pandemic-related stressors*** were measured by asking parents, “Have you been stressed or worried about any of the following as a result of the COVID-19 pandemic in the past 2 weeks?” Options included in the present analyses were financial concerns, relationship challenges, being separated from friends and family, and worrying about the mental health of one’s children. Responses were dichotomized as ‘yes’ or ‘not yes’ (including ‘no,’ ‘don’t know’).

***Pandemic-related coping strategies*** were measured by asking parents, “Which of the following have helped you to cope with stress related to the COVID-19 pandemic in the past 2 weeks?” Options included connecting with those in my household, connecting with my family or friends virtually, maintaining a healthy lifestyle, and going for a walk/exercise.

***Changes to parent–child interactions*** were measured using eight items adapted from previously developed community survey items from the University of Michigan [23]. Parents were asked to indicate how their interactions with their children had been impacted by the COVID-19 pandemic. Example parent–child interactions included having quality time, feeling closeness, or using harsh words. Responses were dichotomized as ‘more’ or ‘not more’ (including ‘less,’ ‘no change’).

***Psychological distress*** was measured using the Kessler-6 item Psychological Distress Scale (K6) [24, 25]. The K6 asks participants to respond to six statements assessing their emotional state during the past 30 days on a scale from 0 (none of the time) to 4 (all of the time). Emotional states included “…so sad nothing could cheer you up,” “nervous,” “restless or fidgety,” “hopeless,” “that everything was an effort,” and “worthless.” Scores ≥ 13 indicate severe psychological distress and scores ≥ 5 < 13 indicate moderate mental distress [26]. Scores on the K6 ≥ 5 were combined into one outcome measure, “moderate to severe psychological distress,” as conducted in previous studies [27, 28]. Internal consistency of this measure in the current sample was good, Cronbach’s α = .90.

***Children’s anxiety*** was measured using the parent-report, brief 8-item Spence Children’s Anxiety Scale (SCAS-P-8) [29, 30]. The SCAS-P-8 asks parents to rate their children’s anxiety levels on a continuous 4-point scale from 0 (never) to 3 (always), across eight items related to separation anxiety, social phobia, and generalized anxiety. Higher scores indicate higher anxiety. There is no set time period over which the judgement has to be made. The SCAS-P-8 has demonstrated evidence of internal consistency, test–retest reliability, convergent and discriminant validity, and capacity to discriminate between children with anxiety disorders (clinical samples) versus community samples [29]. Internal consistency of this measure in the current sample was good, Cronbach’s α = .90.

### Analyses

Analytic sample characteristics were described through proportions and counts. Sociodemographic differences in the proportion of parents reporting moderate to severe psychological distress versus low psychological distress were examined using Chi squared tests.

To address the first objective of identifying sources and mitigating factors of parent psychological distress, logistic regression analysis was conducted to determine the associations between predictor variables and moderate to severe psychological distress. Predictors included parent background characteristics (age, gender, pre-existing mental health conditions, disability), pandemic-related stressors (financial concerns, relationship challenges with a partner, being separated from friends and family, and worrying about the mental health of one’s children), and pandemic-related coping strategies (connecting with those in one’s household, connecting with friends and family virtually or in-person, and going for a walk or exercising outside). All variables were analyzed simultaneously, controlling for each other. A sensitivity analysis was also conducted examining associations with the outcome between each stressor and coping variable independently.

To address the second objective of examining associations between parents’ psychological distress and changes in negative and positive parent-child interactions, separate logistic regression analyses were conducted between parents’ levels of psychological distress (moderate to severe versus low psychological distress) and eight parent-child outcomes including negative interactions (increased discipline, conflicts, harsh words, and yelling/shouting) and positive interactions (increased quality time, feeling closeness, showing love/affection, and observing resilience). Each association was measured independently to account for these types of parent-child interactions being distinct outcomes [23, 31]. Finally, multiple linear regression was conducted to assess the association between parents’ psychological distress and parents’ perceptions of their children’s anxiety. All analyses were adjusted for parent age, gender, pre-existing mental health conditions, and disability.

## Results

Sociodemographic characteristics of the sample are reported in Table 1. A total of 553 participants (49% men, <51% women, <1% non-binary) identified as a parent with a child < 18 years old living at home and were included in the sample. Participants had a mean age of 43 years (SD = 8.5 years). Seventy-seven percent of the parent sample reported having a college education or greater (cf. 67% of adults in Canada according to 2021 census estimates), and 52% reported an annual household income of $100K or more (cf. 40% of adults in Canada according to 2021 census estimates) [32]. Approximately 20% of the sample reported having a pre-existing mental health condition, aligned with pre-pandemic population estimates for adults in Canada [33].

**Table 1:** Sociodemographic characteristics of the parent sample (N = 553).

		Full sample		Proportion within parent subgroups reporting moderate to severe psychological distress^a^	
		N	%	n	%
Gender					
	Men	272	49.2%	144	52.9%
	Women	<280^b^	<50.6%	<171^b^	61.3%
	Non-binary	<5^b^	<0.9%	<5^b^	100.0%
Age					
	18–34	90	16.3%	57	63.3%
	35–54	431	77.9%	247	57.3%
	55+	32	5.8%	13	40.6%
Province					
	Alberta	79	14.3%	51	64.6%
	British Columbia/Territories	62	11.2%	34	54.8%
	Manitoba/Saskatchewan	34	6.1%	27	79.4%
	Atlantic Provinces	43	7.8%	23	53.5%
	Ontario	204	36.9%	122	59.8%
	Quebec	131	23.7%	60	45.8%
Rural/Urban					
	Urban	242	43.8%	149	61.6%
	Rural	106	19.2%	52	49.1%
	Suburban	205	37.1%	116	56.6%
Education					
	High school or less	61	11.0%	37	60.7%
	Some college or university	86	15.6%	54	62.8%
	College or university graduate	406	73.4%	226	55.7%
Marital status					
	Single, never married	35	6.3%	22	62.9%
	Married or partnered	464	83.9%	258	55.6%
	Separated, divorced, widowed	54	9.8%	37	68.5%
Household Income					
	<$50K	73	13.8%	51	69.9%
	$50k to <$100K	180	34.0%	110	61.1%
	$100K +	277	52.3%	143	51.6%
LGBT2Q+					
	Yes/Unsure	33	6.0%	21	63.6%
Pre-existing mental health					
	Yes	113	20.4%	93	82.3%
Disability					
	Yes	45	8.1%	35	77.8%
Visible minority					
	Yes	200	36.2%	115	57.5%
Household living situation (check all that apply)					
	Living with spouse/partner	449	81.2%	247	55.0%
	Living with other adult family	25	4.5%	20	80.0%
	Living with grandchildren	6	1.1%	<5^b^	<83.0%
Age of children at home (check all that apply)					
	< 4	128	23.1%	84	65.6%
	5–11	266	48.1%	158	59.4%
	12–17	295	53.3%	157	53.2%
	18 +	67	12.1%	28	41.8%
Child siblings at home					
	Yes	292	52.8%	177	60.6%

^a^Moderate to severe psychological distress measured as scores on the K6 ≥ 5

^b^Exact number suppressed due to small cell size for the sake of anonymization.

### Role of parent characteristics, stressors, and coping strategies predicting parent psychological distress

The mean level of psychological distress for parents in the full sample was 6.72 (SD = 5.45) as measured on the K6, indicating that on average, parents in this sample met the criteria for moderate psychological distress. Overall, 57% of parents reported moderate to severe psychological distress. In unadjusted analyses, moderate to severe psychological distress was disproportionately reported by parents with pre-existing mental health conditions, parents with disabilities, parents reporting financial concerns, and parents with younger children < 4 years old at home (S2 Table).

Table 2 presents adjusted associations between parent characteristics, pandemic-related stressors, and coping strategies predicting parent psychological distress. Having a pre-existing mental health condition was associated with nearly four times the odds of moderate to severe psychological distress, even after accounting for age, gender, disability, stressors, and coping strategies. Adjusting for all other variables, parents who reported financial concerns, relationship challenges with one’s partner, stress due to worrying about being separated from friends and family, and stress due to worrying about the mental health of one’s children, had up to three times the odds of moderate to severe psychological distress. Parents who reported going for a walk/exercise outside as a way to cope with stress due to the pandemic had significantly lower odds of severe psychological distress compared to those who did not report these strategies, again adjusting for all other variables. A sensitivity analysis examining each stressor and coping variable independently, while adjusting for parent characteristics, showed the same pattern of associations.

**Table 2:** Role of parent characteristics, stressors, and coping strategies predicting parent psychological distress during the COVID-19 pandemic.

		Parent Moderate to Severe Psychological Distress		
		OR	95% CI	
Parent characteristics*				
	Age < 35	1.01	0.57	1.82
	Women	1.19	0.78	1.80
	Pre-existing mental health condition	**3.71**	**1.98**	**6.94**
	Disability	1.13	0.46	2.80
**Stressors**				
	Financial concerns	**1.73**	**1.10**	**2.72**
	Experiencing relationship challenges with my partner	**2.54**	**1.47**	**4.41**
	Being separated from friends and family	**3.12**	**1.90**	**5.13**
	Worrying about how the mental health of my child(ren) will be affected by the pandemic	**1.65**	**1.05**	**2.59**
**Coping Strategies**				
Connecting with those in my household		0.91	0.58	1.43
Connecting with friends and family virtually		0.71	0.44	1.14
Connecting in-person with friends or family		0.70	0.44	1.12
Going for a walk/exercise outside		**0.64**	**0.42**	**0.97**

All variables have been analyzed simultaneously. Reference group is parents reporting low psychological distress, K6 < 5. Bold indicates statistically significant associations.

### Role of parent psychological distress predicting parent-child interactions and child anxiety

In the overall sample, 12% of parents reported disciplining their children more frequently due to the pandemic, 18% reported more conflicts with children, 10% reported more frequent use of harsh words, and 12% reported more yelling and shouting at their children. At the same time, 47% of parents reported having more quality time with their children, 37% reported feeling more closeness with their children, 37% showing more love and affection to their children, and 31% reported observing resilience more frequently in their children due to the pandemic. Table 3 presents eight separate associations between parents’ levels of psychological distress and changes in their interactions with their children (four negative changes and four positive changes), adjusting for parent age, gender, pre-existing mental health conditions, and disability. In the adjusted models, parents reporting moderate to severe psychological distress had more than double the odds of reported increased negative parent-child interactions due to the pandemic, including nearly seven-times the odds of using harsh words with their children (Table 3). Moderate to severe psychological distress was not associated with increases in positive parent-child interactions (Table 3).

**Table 3:** Role of moderate to severe parent psychological distress predicting changes in parent-child interactions during the COVID-19 pandemic^a^.

			OR		95% CI		
**Negative parent-child interactions**							
	Disciplining my child(ren)	**2.66**		**1.45**		**4.88**	
	Conflicts with my child(ren)	**2.75**		**1.63**		**4.66**	
	Using harsh words with my child(ren)	**6.91**		**2.87**		**16.64**	
	Yelling/shouting at my child(ren)	**4.71**		**2.32**		**9.58**	
**Positive parent-child interactions**							
	Having more quality time with my child(ren)		1.16		0.81		1.66
	Feeling closeness with my child(ren)		1.22		0.84		1.76
	Showing love or affection to my child(ren)		1.39		0.96		2.01
	Observing resilience (strength and perseverance) in my child(ren)		1.33		0.89		1.97

Odds ratios show the association between parent psychological distress and increased frequency of parent-child interactions due to the COVID-19 pandemic.

^a^Each association has been analyzed separately, adjusting for parent age, gender, pre-existing mental health condition, and disability. Reference group is parents reporting low psychological distress, K6 < 5. Bold indicates statistically significant associations.

The mean children’s anxiety score reported by parents in this sample was 4.23 (SD = 4.25) as measured by the SCAS-P-8. After adjusting for parent age, gender, pre-existing mental health condition, and disability, parents who reported moderate to severe psychological distress reported their children’s anxiety to be 2.81 points higher (95% CI = 2.21, 3.50) compared to parents not reporting moderate to severe psychological distress.

## Discussion

This study examined the prevalence of parent psychological distress in Canada, using data from nearly two years into the COVID-19 pandemic. Related aims were to identify sources and mitigating factors of parents’ psychological distress, and associations between parents’ psychological distress and impacts on family life including parent-child interactions and perceptions of children’s anxiety. Overall, 57% of parents in this sample reported moderate to severe psychological distress—nearly double pre-pandemic population levels of psychological distress reported among adults in North America using the same K6 measure [26]. Our analysis adds to the evidence identifying parents and children as groups particularly impacted by the pandemic. Importantly, our findings extend previous insights identifying sustained distress and disrupted family functioning nearly two years into the pandemic. The implications are that long-term, systemic responses are needed that address underlying sources of psychological distress for families and improve the availability of mental health supports for parents and their children.

Other research worldwide has similarly identified increases in population psychological distress during the COVID-19 pandemic specifically among parents [9, 10, 34]. One study in Japan reported parents’ distress to be double pre-pandemic national estimates in May 2020 during a state of national emergency and school closures [35]. Concerningly, results from our study in Canada suggest that parents continued to report elevated and sustained psychological distress nearly two years later, at a time when COVID-19 vaccination rates were high and infection rates were trending downward [20]. These findings may support concerns of psychological ‘scarring’ or prolonged distress, with mental health impacts of the pandemic enduring beyond the first physical health threats of the virus. The current study also identified social inequities in psychological distress consistent with disparities observed during the first and third waves of the COVID-19 pandemic, where parents with pre-existing mental health conditions, parents with disabilities, parents reporting financial strain, and parents with younger children reported higher levels of psychological distress than other population groups [2, 8, 10]. In the present study, after adjusting for parent age, gender, pre-existing mental health conditions, disability, and coping strategies, parents experiencing stressors including financial concerns, relationship challenges, separation from friends and family, and worries about their children had 1.5 to 3 times the odds of moderate to severe psychological distress. These results highlight that the inequitable impacts of the pandemic were still being felt two years later, and that risks of psychological distress were heightened for parents who were living in conditions that were more likely to exacerbate these stressors.

In the overall sample, parents reported increases in negative parent-child interactions due to the pandemic (10% more frequent harsh words, 12% more discipline and yelling, 18% more conflicts). The proportion of parents reporting pandemic-related increases in negative parent-child interactions nearly two years into the pandemic was slightly less compared to the proportion of parents reporting increases in negative parent-child interactions in a separate cross-sectional sample in Canada at the beginning of the pandemic [2]. Consistent with earlier pandemic research and family stress theories [13–15, 36], parents in the current study who reported moderate and severe psychological distress had higher odds of reporting increased use of discipline, conflict, harsh words, and yelling with their children.

Parents also reported increases in positive parent-child interactions due to the pandemic (31% observing resilience, 37% feeling more closeness and showing more love and affection, 47% reporting more quality time). This result is consistent with previously reported increases in positive parent-child interactions during the pandemic, perhaps as a result of more opportunities to spend time together [2, 8]. Again, the proportion of parents reporting pandemic-related increases in positive parent-child interactions nearly two years into the pandemic was slightly less compared to the proportion of parents reporting increases in positive parent-child interactions in a previous cross-sectional sample at the beginning of the pandemic [2]. In the current study, parents’ psychological distress was not associated with increases in positive parent-child interactions. Overall, the results suggest that changes in parent distress levels during this period of the COVID-19 pandemic may not have adversely affected positive family functioning in the same way it adversely affected negative family functioning.

In terms of protective factors for families, parents who reported going for walks outside as a strategy for coping with stress due to the pandemic had lower odds of moderate to severe psychological distress. However, this strategy also highlights disparities in social and structural resources available to families, which might further exacerbate mental health inequities over time. For example, going for a walk outside necessitates access to a safe and appropriate outdoor space, a resource that has been associated with mental health benefits during the pandemic [37–40]. Future research should investigate inequities in positive coping strategies as they relate to disparities in access, as well as the uptake of negative coping strategies in response to stress from the pandemic, such as alcohol use. Moving forward, these and other inequities should be considered and supports prioritized among parent populations who are disproportionately experiencing low social support and lack of access to spaces and amenities that promote well-being.

Lastly, there has been widespread concern that parent distress and disruptions to family functioning due to the pandemic may also exacerbate outcomes for children including increased rates of anxiety and depression [13]. While we were unable to assess a directional or causal relationship due to the cross-sectional design of our study, the use of a validated and commonly used parent-reported children’s anxiety scale enabled us to observe that parents who reported moderate and severe psychological distress perceived their children’s anxiety to be significantly higher compared to parents who reported low distress. This could be due to parents and children’s distress both increasing as a result of common pandemic-related stressors (e.g., prior research identifies that periods of uncertainty with indeterminant endpoints are particularly stressful for children [41–43]). It could also be that increases in parent distress and subsequent harsh parenting and conflict led to increased anxiety among children of more distressed parents, aligned with the family stress model [15] and supported by research on changes to family functioning during the COVID-19 pandemic [13, 44]. Future studies could further elucidate these pathways between stressors and parent and child mental health.

### Strengths and limitations

This study was based on a large, nationally representative sample, providing the ability to examine inequities in psychological distress outcomes by social and structural stratifications within the population. We drew on previously validated and commonly used measures of psychological distress (K6) [24] and children’s anxiety (Spence Children’s Anxiety Scale) [28, 29], which enabled comparisons with pre-pandemic samples and also allows for future comparisons to be made with other population studies.

Limitations of this study must be considered when interpreting the results. Due to the cross-sectional nature of this study, we cannot determine the direction of association or causality between psychological distress, stressors, coping mechanisms, parent-child relationships, and children’s anxiety. Parents with greater psychological distress may be more likely to perceive distress in their children [45], and relatedly, potentially perceive worsened relationships with their children [46]. However, in regards to parent-child relationships, the lack of association between parent distress and changes in positive parent-child interactions suggests that our results may not have been substantially affected by reporting bias. We also did not have pre-pandemic baseline measures of psychological distress or child mental health for parents in this sample, which limited the conclusions we could draw regarding changes in parent-child relationships or mental health due to the pandemic. However, previous pandemic research that has used the K6 and included baseline distress measures has similarly identified increases in parent psychological distress, with approximately 7% of respondents reporting newly developed depressive and anxiety symptoms due to the pandemic [9]. Future research could better illuminate these patterns and associations by linking parents’ data to their children’s self-reported mental health or administrative data, and by including baseline measures of parents and children’s mental health. Our data should also be interpreted in the wider social-ecological context of the pandemic experience in Canada. Factors due to the timing of data collection and political climate such as COVID-19 infection rates, seasonality, lockdowns, vaccination rates, and available social safety nets such as financial supports may not be generalizable to other contexts.

Oversampling and recruitment from community organizations were strategies employed to overcome common limitations of online survey data collection, including low representation from populations who face technological barriers. The demographic composition of our sample indicates that respondents were more likely to be higher educated, white, and higher income. Underrepresentation of racialized parents, and parents on the lower end of the socioeconomic spectrum, may have underestimated the prevalence of psychological distress reported in this study, as previous research has indicated these population groups have experienced disproportionately worse mental health due to the pandemic [46, 47]. It is also possible that parents experiencing greater distress may have less time, opportunity, or desire to respond to an online survey, again potentially underestimating results reported in this study.

## Conclusions

This study identifies that parents with children < 18 years of age living at home in Canada were experiencing heightened psychological distress nearly two years into the pandemic. Concerningly, parents who reported moderate to severe psychological distress also reported increases in discipline, conflict, harsh words and yelling with their children, and perceived heightened anxiety among their children. These findings add to the existing body of literature suggesting that negative impacts of the pandemic on family functioning might extend beyond the first physical health threats of the virus. We hope these findings will inform universal and targeted public health and policy initiatives necessary to mitigate the widening mental health disparities among families, particularly among those at a disproportionate risk for social and health inequities. The longitudinal impacts of the pandemic on family functioning must be continue to be monitored, including impacts on parent-child relationships and adults’ and children’s mental health, with timely interventions that support well-being for parents and their children.

## Supporting information

S1 Table Study survey items. (PDF)

S2 Table Proportions of parents with moderate to severe versus low psychological distress during the COVID-19 pandemic by sociodemographic factors. (PDF)
