# Supplementary material for: Parent psychological distress and parent-child relationships two years into the COVID-19 pandemic: Results from a Canadian cross-sectional study
Source: PLoS One. 2023 Oct 17;18(10):e0292670. doi: 10.1371/journal.pone.0292670 (PMC10581480; doi:10.1371/journal.pone.0292670)
Supplement: S1 Table — (PDF) [file pone.0292670.s001.pdf]

Supplementary S1 Table. Study survey items

Kessler-6 Psychological Distress Scale

The following questions ask about how you have been feeling during the past 30 days. For each question, please indicate how often you have this feeling.

During the past 30 days, how often did you feel…<br>1. …nervous;<br>2. …hopeless;<br>3. …restless or fidgety;<br>4. …so depressed that nothing could cheer you up;<br>5. …that everything was an effort;<br>6. …worthless.	Response options:<br>All of the time<br>Most of the time<br>Some of the time<br>A little of the time<br>None of the time

PARENT PSYCHOLOGICAL DISTRESS

Stressors

Have you been stressed or worried about any of the following as a result of the COVID-19 pandemic in the past 2 weeks? (Please select one option on each row)

1. Financial concerns (e.g. going into debt, ability to pay bills, long-term economic impacts, etc.)<br>2. Experiencing relationship challenges with my partner<br>3. Being separated from friends and family<br>4. Worrying about how the mental health of my child(ren) will be affected by the pandemic	Response options:<br>Yes<br>No<br>Don’t know /Not applicable/ Prefer not to say

Coping

Which of the following have helped you to cope with **<u>stress</u>** related to the COVID-19 pandemic in the **past 2 weeks**? (Please select all that apply)

1. Connecting with those in my household<br>2. Connecting with my family or friends virtually (e.g., phone, video chat, etc.)<br>3. Connecting in-person with friends or family<br>4. Going for a walk/exercise outside	Response options:<br>Yes<br>No

PARENT PSYCHOLOGICAL DISTRESS

Parent-Child Interactions

Please indicate how your interactions with your child(ren) have been impacted by the COVID-19 pandemic. (Please select one option on each row)

1. Having quality time with my child(ren)<br>2. Feeling closeness with my child(ren)<br>3. Showing love or affection to my child(ren)<br>4. Observing resilience (strength and perseverance) in my child(ren)<br>5. Disciplining my child(ren)<br>6. Conflicts with my child(ren)<br>7. Using harsh words with my child(ren)<br>8. Yelling/shouting at my child(ren)	Response options:<br>More<br>Less<br>No change<br>Not applicable<br>Prefer not to say

PARENT PSYCHOLOGICAL DISTRESS

Brief Spence Children's Anxiety Scale (P-8)

Below is a list of items that describe children. For each item please circle the response that best describes your child. Please answer all the items. There is no set time period over which the judgement has to be made.

1. My child worries that something bad will happen to him/her<br>2. My child complains of feeling afraid<br>3. All of a sudden my child feels really scared for no reason at all<br>4. My child worries about being away from us / me<br>5. My child worries what other people think of him/her<br>6. My child has trouble going to school in the mornings because (s)he feels nervous or afraid<br>7. My child feels afraid that (s)he will make a fool of him/herself in front of people<br>8. My child worries about things	Response options:<br>Never<br>Sometimes<br>Often<br>Always
