# Supplementary material for: Parent psychological distress and parent-child relationships two years into the COVID-19 pandemic: Results from a Canadian cross-sectional study
Source: PLoS One. 2023 Oct 17;18(10):e0292670. doi: 10.1371/journal.pone.0292670 (PMC10581480; doi:10.1371/journal.pone.0292670)
Supplement: S2 Table — (PDF) [file pone.0292670.s002.pdf]

Supplementary S2 Table. Proportions of parents with moderate to severe versus low psychological distress during the COVID-19 pandemic by sociodemographic factors

	**GENDER**		**AGE**		**PRE-EXISTING MENTAL HEALTH CONDITION**		**DISABILITY**		**LGBT2Q+**		**PARENT TO A CHILD <4 YEARS OLD**		**FINANCIAL CONCERNS**	
	Women (n=279)	Men (n=272)	<35 years (n=90)	35+ years (n=463)	Yes (n=113)	Not yes (n=440)	Yes (n=45)	Not yes (n=508)	Yes (n=33)	No (n=520)	Yes (n=128)	No (n=425)	Yes (n=207)	No (n=346)
Moderate to severe psychological distress	171<br>61.3%	144<br>52.9%	57<br>63.3%	260<br>56.2%	93<br>82.3%**	224<br>50.9%	35<br>77.8%*	282<br>55.5%	21<br>63.6%	296<br>56.9%	84<br>65.6%*	233<br>54.8%	157<br>75.8%**	160<br>46.2%

**p*<.05, ***p*<.001

Reference group is parents reporting low psychological distress K6 < 5. Proportions compared using Chi squared tests.
